# Domestic Water Service Delivery Indicators and Frameworks for Monitoring, Evaluation, Policy and Planning: A Review

**DOI:** 10.3390/ijerph10104812

**Published:** 2013-10-11

**Authors:** Georgia L. Kayser, Patrick Moriarty, Catarina Fonseca, Jamie Bartram

**Affiliations:** 1 The Water Institute, Gillings School of Global Public Health, University of North Carolina at Chapel Hill, Chapel Hill, NC 27599, USA; E-Mail: gkayser@unc.edu; 2 IRC International Water and Sanitation Centre, Bezuidenhoutseweg 2, The Hague 2594 AV, The Netherlands; E-Mails: moriarty@irc.nl (P.M.); fonseca@irc.nl (C.F.)

**Keywords:** drinking water services, water indicators and frameworks, monitoring & evaluation, policy & planning, public health, human rights, poverty reduction

## Abstract

Monitoring of water services informs policy and planning for national governments and the international community. Currently, the international monitoring system measures the type of drinking water source that households use. There have been calls for improved monitoring systems over several decades, some advocating use of multiple indicators. We review the literature on water service indicators and frameworks with a view to informing debate on their relevance to national and international monitoring. We describe the evidence concerning the relevance of each identified indicator to public health, economic development and human rights. We analyze the benefits and challenges of using these indicators separately and combined in an index as tools for planning, monitoring, and evaluating water services. We find substantial evidence on the importance of each commonly recommended indicator—service type, safety, quantity, accessibility, reliability or continuity of service, equity, and affordability. Several frameworks have been proposed that give structure to the relationships among individual indicators and some combine multiple indicator scores into a single index but few have been rigorously tested. More research is needed to understand if employing a composite metric of indicators is advantageous and how each indicator might be scored and scaled.

## 1. Introduction

There are public health, economic and human rights-related benefits from access to a sufficient supply of safe drinking water. In 2010 a United Nations General Assembly resolution recognized that a sufficient and safe supply of water is a human right and is essential for the realization of many other human rights. A similar logic influenced the inclusion of access to sustainable, safe drinking water in the Millennium Development Goal (MDG) target 7.C to “halve, by 2015, the proportion of the population without sustainable access to safe drinking water…” and in earlier development initiatives since the 1960s [1].

To measure progress toward target 7.C, data have been analyzed using a binary categorization of households as using “improved” or “unimproved” drinking water sources. “Improved” drinking water sources include: piped water to the dwelling, plot or yard, public taps, tubewell/borehole, protected dug well, protected spring, or rainwater. “Unimproved” drinking water sources include: an unprotected dug well, unprotected spring, cart with tank or drum, tanker truck, surface water, or bottled water [2]. While useful in identifying and communicating trends in water supply, this system does not fully assess water services for purposes such as public health protection, human rights fulfillment, and poverty reduction. This would require insight into specific factors such as water safety, quantity, accessibility, reliability/continuity, equity, and affordability of water provision.

The MDG target for water was declared to have been met in 2012; yet, this announcement has been criticized for its failure to reflect the safety and sustainability dimensions of the target [3]. The result is less accountability for and transparency in the public health, economic and human rights benefits of the water service [4,5,6,7,8,9,10]. The use of multiple, individually more nuanced indicators to describe water services may contribute to overcoming these shortcomings and assist in the monitoring of water services, internationally and in low, middle and high income countries.

Indicators are used to guide policy formulation, frame international monitoring and may catalyze civil society as epistemic communities influence policy decisions [11,12]. A panel of indicators, each measured along a scale, may reflect different degrees of benefits, and may contribute to accountability as epistemic communities create norms, assist in analyzing progressive realization of the human right to water and help track improvements to public health protection [11,12,13,14,15,16]. The human rights community has also embraced indicators as part of a goal to have assessment and monitoring of state obligations for the realization of human rights [17]. Furthermore, in 2012–2013, the World Health Organization (WHO) and United Nations Children’s Fund (UNICEF), under the Joint Monitoring Programme (JMP), made concerted efforts to identify targets and indicators for global sector post-2015 monitoring [18].

Water service monitoring frameworks provide a set of indicators that can be used to track trends and measure progress. Comparisons can be made across countries, between service providers and among technologies. Ideally, the desired public health, economic and human rights benefits respond to incremental improvement in each indicator. A framework identifies the elements (indicators) and general relationships among these elements that one needs to consider for institutional analysis [19]. A framework provides a general set of variables (in this case indicators) that can be used to analyze institutional arrangements [19]. 

A water service index combines a set of measurable indicators of a water service into a single measure. A single composite metric may be achieved, for example, by combining the values of the indicators along comparable scales so as to determine a single level of service. Conceptualizing service levels as rungs of a ladder provides a useful metaphor for incremental progress [5,20]. 

The goal of monitoring water services is to contribute to the extension of and improvement in service delivery, thereby, securing the public health, economic, and human rights benefits that are sought from improved water supply [21]. Monitoring water services can thus contribute to global and national policies and influence planning.

This paper provides a history of development of water service monitoring indicators, the frameworks into which they have been organized, and indices derived from them. It is intended to contribute to the policy debate about future global water goals targets and indicators. The most frequent indicators are examined and the relationships of each to public health, economic development, and human rights reviewed. The advantages and disadvantages of using a framework or index are discussed in the context of contemporary debate on post-MDG international development policy and associated monitoring, including their role in planning or evaluating national and international water services.

## 2. Frameworks for Monitoring Water Services: A History

The monitoring of domestic water supply that incorporates an approach whereby multiple indicators, each on a scale of acceptability instead of a binary approach of “haves” and “have-nots” can be traced back to 1991 [5]. Section 2.1 through Section 2.6 relay the history of domestic water service frameworks that were developed between 1991 and 2011. Monitoring of certain indicators can be traced back long before 1991. For example, in 1972, White, Bradley and White’s study of household water supply and productivity in East Africa, concluded, “the way people respond to present and improved supplies and the effect this has on community health and welfare should be examined for the whole range of theoretically possible improvements” [22]. The use of frameworks to understand the quality of a service provided across a number of indicators, however, began in 1991 and is reviewed in Section 2.

### 2.1. Measuring and Ranking Water Services along Indicators *(*1991 and 1996*)*

**Figure 1:**
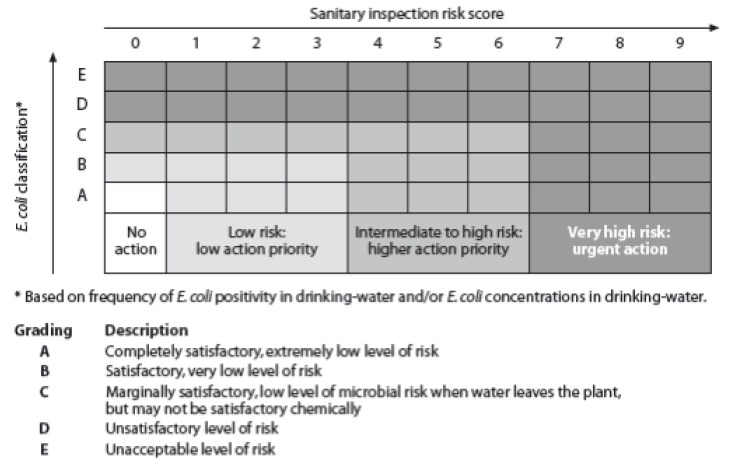
A Framework for Monitoring Water Quality & Sanitary Risk (Reprinted from Water Science and Technology **1991**, volume *24*, pages 61–75, with permission from the copyright holders, IWA Publishing).

In 1991, Lloyd and Bartram introduced a health risk based approach to water service monitoring that went beyond a simple pass/fail approach [23]. They asserted that “the focus on increased coverage needs to be amplified to include improvement of the quality of service” and proposed a surveillance strategy for progressive improvement of water services based on a framework of indicators [23]. They defined water service levels based on multiple indicators of service quality related to potential health risk. They proposed a framework of five indicators each on a scale rather than as a single pass/fail benchmark. The metrics to gauge health risk included: (1) coverage, measured by the supply type; (2) continuity, measured by hours per day and days per year that water is supplied; (3) quantity, measured by volume supplied per capita; (4) sanitary risk (measured by an *E. coli* count scale (from >1,000 *E. coli*/100 mL at the highest risk to <1 *E. coli*/100 mL at no risk) combined with sanitary inspection), and (5) cost, measured by tariff paid per month per household [23]. The approach was found to have robust results in Peru, and was later adapted by the WHO *Guidelines for Drinking-water Quality* [24,25]. These guidelines suggested that the performance of water supply systems should be evaluated along five factors: quality, coverage, quantity, continuity, and cost [24]. Figure 1 is a graphic depiction of how sanitary risk and water quality is measured. Sanitary inspection score (0–9) across the x-axis reflects progressively higher sanitary risk as the score increases from 0 to 9 and is scored in response to a sanitary survey. The *y*-axis represents *E. coli* presence from high to low risk from A to E. 

In 1996, Bartram proposed a simplification of the five component approach, arguing that there was overlap among some of the indicators [26]. Specifically, Bartram suggested that a service level is conditioned by two factors: continuity and quality (safety), such that service quality = service level × continuity × safety in which inadequate continuity or quality reduce the value of the service level [26]. This is reflected in contemporary thinking in linking the two concepts of provision (service level attained) and risk (factors which undermine the value of the service level or impede its enjoyment) [27].

### 2.2. Water Service Levels Introduced *(*2003*)*

In 2003, Howard and Bartram proposed a series of benchmarked water service levels in response to calls for information on water quantity, access and service level impacts on health [9]. They categorized water service levels along a scale of linked indicators that included: water quantity and water source accessibility [9] (Table 1). Working from a public health perspective, each increase in level (from no access to optimal access) was linked to a decrease in associated health risk. Water quantity for adequate hydration and hygiene were emphasized in the water service levels developed. This reflected research that found a rapid decrease in water consumption as travel time to the water source increases [22,28], and a decrease in hygiene, specifically handwashing, in households with diminished water quantity available [29]. Howard and Bartram’s approach also mentioned the health benefits derived from improved nutrition and food security from the increased availability of water for use on small garden plots, reflecting the work of Tompson *et al*. [30].

**Table 1:** Water service levels (Reprinted from [9] with permission).

Service level	Access	Needs met	Level of health concern
No access (quantity collected often below 5 L/c/d)	More than 1,000 m or 30 min total collection time	Consumption—cannot be assured Hygiene—not possible (unless practiced at source)	Very high
Basic access (average quantity unlikely to exceed 20 L/c/d)	Between 100 and 1,000 m or 5 to 30 min total collection time	Consumption—should be assured Hygiene—hand washing and basic food hygiene possible; laundry/bathing difficult to assure unless carried out at source	High
Intermediate access (average quantity about 50 L/c/d)	Water delivered through one tap on plot (or within 100 m or 5 min total collection time	Consumption—assured Hygiene—all basic personal and food hygiene assured; laundry and bathing should also be assured	Low
Optimal access (average quantity 100 L/c/d and above)	Water supplied through multiple taps continuously	Consumption—all needs met Hygiene—all needs should be met	Very low

### 2.3. A Human Rights Framework for Water *(*2003, 2010*)*

In 2010, the United Nations General Assembly and United Nations Human Rights Council recognized water and sanitation as a human right [31,32]. The human right to water had been described by General Comment 15 in 2003 [32], defining the parameters of normative concern. *The UN-recognized* “The Human Right to Water and Sanitation”, is met through progressive realization of universal access to sufficient, safe, physically accessible, and affordable water [31,32]. The UN Special Rapporteur on the Human Right to Safe Drinking Water and Sanitation noted that the MDGs do not sufficiently address equity and non-discrimination, and emphasized that indicators and their monitoring are critical to operationalizing the human right to water and sanitation, providing precision in state reporting and structuring a human rights based approach to water [8]. The United Nations Committee on Economic, Social and Cultural Rights (UNCESCR), therefore, identifies non-discrimination and equality as fundamental principles [32] (Table 2). 

**Table 2:** The right to water (Adapted from [*General Comment No. 15: The Right to Water*]*.* by UNCESCR, copyright (2003) United Nations. Reprinted with permission of the United Nations).

Indicator	Definition
**Availability**	The water supply for each person must be sufficient and continuous for personal and domestic uses. These uses ordinarily include drinking, personal sanitation, washing of clothes, food preparation, personal and household hygiene. According to the WHO, between 50 and 100 L of water per person per day are needed to ensure basic needs are met and few health concerns arise [29].
**Quality**	The water required for personal or domestic use must be safe, therefore free from micro-organisms, chemical substances and radiological hazards that constitute a threat to a person’s health. Measures of drinking-water safety are usually defined by national and/or local standards for drinking-water quality. The World Health Organization (WHO) *Guidelines for Drinking-water Quality* provide a basis for the development of national standards.
**Accessibility**	Water facilities must be accessible to everyone without discrimination. Accessibility has overlapping dimensions: physical, economic, and information. Sufficient and safe water must be accessible within the vicinity of the household and affordable. According to WHO, the water source has to be within 1,000 m of the home and collection time should not exceed 30 min. The United Nations Development Programme (UNDP) suggests that water costs should not exceed 3 per cent of household income. Accessibility includes the right to seek, receive and impart information concerning water issues.
**Non-discrimination and equality**	It is the obligation of States to guarantee that the right to water is enjoyed without discrimination and equally between men and women and proscribes any discrimination which has the effect of nullifying or impairing the equal enjoyment or exercise of the right to water.

This framework, outlined in the right to water, has not been field-tested nor have scales been allocated to each indicator incorporated into the framework.

### 2.4. The WHO-UNICEF Framework *(*2008 et seq.*)*

The WHO has been collecting data on drinking water services since 1962. In 1962, data was collected from urban areas in 75 countries on the percentage of households served with piped water to the house or from public standpipes [33]. In 1972, data collection expanded to rural areas, new data was added on distance to source, and data was collected in 96 countries. Reasonable access was considered use of water less than 200 m from the household [33]. The WHO continued to collect this data and in 1990 joined with UNICEF to establish the JMP to monitor national progress toward universal access to safe drinking water and sanitation. In 2000, there was a shift in method in that data from censuses and nationally representative household surveys, rather than that reported by national authorities, were the primary data sources [33]. 

**Figure 2:**
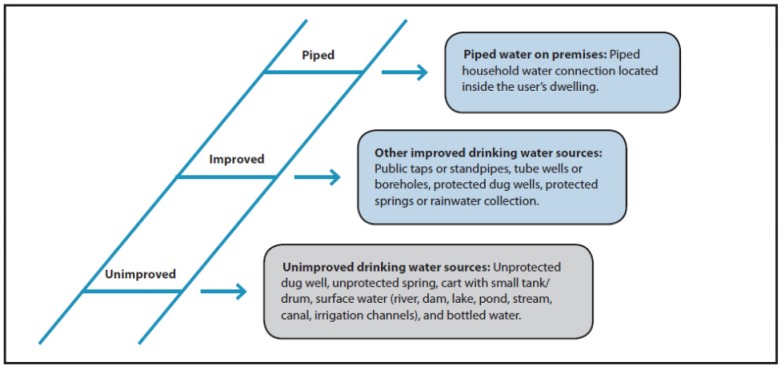
The WHO-UNICEF JMP water serivce ladder (Adapted from [34] with permission).

In 2008, the term “water service ladder” was introduced to international monitoring by the World Health Organization (WHO) and the United Nations Children’s Fund (UNICEF) in a Joint Monitoring Program (JMP) water and sanitation Millennium Development Goal (MDG) progress report to describe the distribution of different qualities of drinking water access across and between populations [25]. The water service ladder comprised three rungs and was based on the type of sources which households used (Figure 2). On the bottom rung were “unimproved” water sources, with “improved” water sources on the middle rung, and “piped water on the premises,” piped to the household dwelling, plot or yard, on the top rung [34]. There is a long history of technology categorization driven assessment and the rungs of the ladder have in fact been reported on for around half a century, but the ladder metaphor was new to international drinking water monitoring in 2008.

This is the only water service framework that is supported by data collection at scale, throughout the world, and that can be aggregated and disaggregated at different geographical scales.

### 2.5. A Framework for Multiple Use Services *(*2007 and 2009*)*

**Figure 3:**
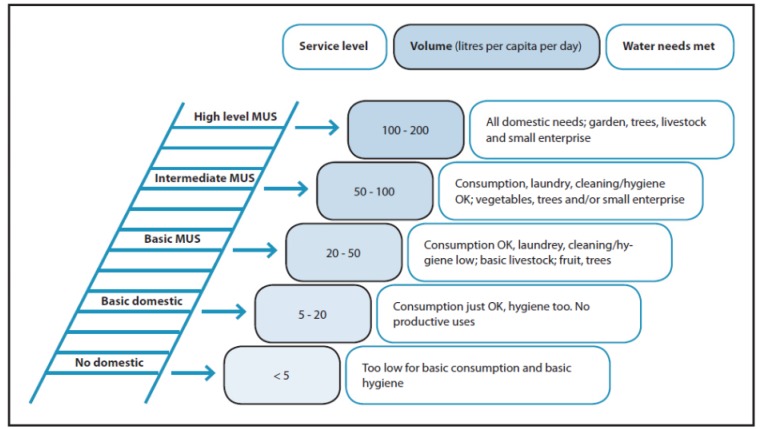
A multiple-use services framework (Adapted from [35] with permission).

In 2007, in parallel development, Renwick *et al*. used water service levels in their work on Multiple-Use Services (MUS) to assess one indicator, water quantity, along a continuum, which included livelihood and productive uses [35]. The MUS ladder incorporated indicators of distance, volume, and a description of main water uses that a given quantity of water could support (see Figure 3). They described MUS as “a participatory integrated and poverty-reduction focused approach in poor rural and peri-urban areas, which takes people’s multiple water needs as a starting point for providing integrated services” [36]. The MUS service level approach added water for productive household needs to that for hygiene and drinking, introduced indicators for livelihood or productive use (domestic and productive uses), to the water service ladder, and quantified the level of productive household activities that a given quantity of water can support. Using a MUS framework, water service levels are ranked according to the quantity of water available to meet demands for drinking, hygiene, bathing, laundry, cleaning, gardening, livestock, irrigation, and small enterprise. Using similar logic, in 2009, van Koppen *et al*. put forward a MUS ladder that built on the work of Howard and Bartram (2003) and Renwick *et al*. (2007) and focused on economic and poverty-reduction benefits [9,35,36,37].

The framework and the quantities allocated for different water needs was created from a literature review, field observations and through interviews with experts [35]. The MUS framework has been tested in Asia and sub-Saharan Africa to understand the costs and benefits of using single *vs*. multiple use services with a strong focus on rural areas and agriculture [35]. The MUS framework itself, however, has not been field tested to determine if the needs met at each quantity of water or service level are correct for a variety of geographical areas.

### 2.6. Water Service Ladders *(*2008 and 2009*)*

**Figure 4:**
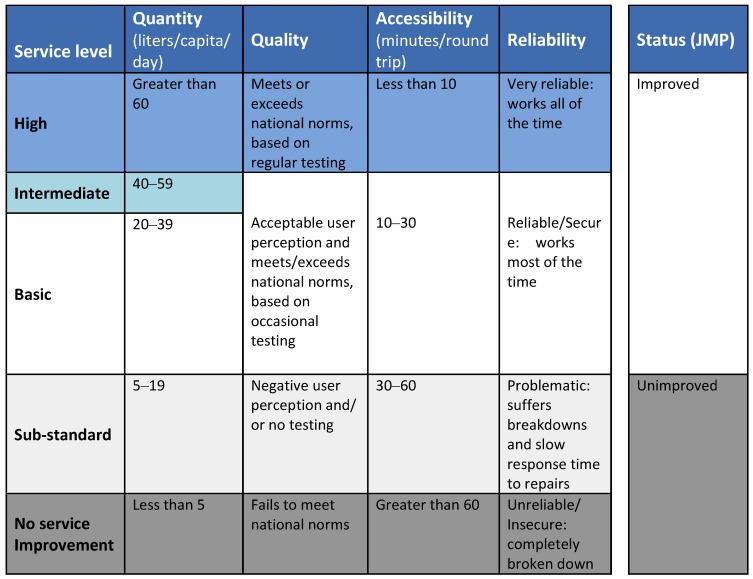
IRC water service delivery ladder framework (Adapted from [20,38] with permission).

In 2008 and 2009, the International Water and Sanitation Center (IRC) continued the ladder metaphor and developed a five-rung domestic water service delivery ladder ranging from “no-service” to high service level. The IRC ladder is based on categorizations of four indicators: quantity, quality, accessibility and reliability (see Figure 4). For example, the “quality” indicator was assessed as “unacceptable”, “acceptable” or “high”, based on biological and chemical measures to determine a level. The IRC ladder allowed for a single measure of the service of each household by assigning a service level category (no-service to high level service) based on the value of the lowest (worst) indicator measured. The MDG JMP water status indicator was included for comparison.

This framework was based on a review of sector wide thinking over the previous two decades [20,38]. It was created to measure the quality of water services that households had access to so that the cost of the service could be estimated [20,38]. It was developed as part of an effort to shift away from costing and counting water supply hardware and towards measuring the quality of the service delivered to the user over time. The development and use of the ladder was also motivated by a desire to influence the policy debate, raise awareness about service delivery that went beyond hardware, and to provide a language and tools for measuring service delivery. As such, selection of indicators and ranges was strongly driven by pragmatic consideration of existing policy and norms, internationally and in the countries where IRC was working. The ranges were determined by interviews with experts and national policy makers. The ladder presented in Figure 4 is a composite international ladder. IRC ladders used in countries could and did differ in detail, based on local context. 

This service ladder was tested in Ghana, Burkina Faso, Mozambique and India (Andhra Pradesh). The ladder was adapted in each country to national norms and standards. This approach identified (among others) both high levels of non-functionality and poor service delivery [37,38,39,40,41,42,43]; and wide ranges of costs in achieving (or failing to achieve) targeted levels of service. A strength of the IRC water service ladder is its flexibility to adapt to national norms and standards. This, however, is also a weakness as it makes comparisons across countries difficult. 

## 3. Water Service Indicators

It is important to understand the value of each of the indicators employed in the previous monitoring frameworks. These include water source/technology type (including whether categorized as “improved,” “unimproved,” community source, or on-plot water), accessibility, water safety (quality and sanitary risk), water quantity, reliability or continuity, affordability, and equity.

Here we review the public health, economic, and rights-based justifications, realized in research and practice, for their use in monitoring and evaluation, policy and planning. Frameworks then serve to explain the relationships among the various indicators.

### 3.1. Service Type (Infrastructure Classification)

Service types are sometimes referred to as water sources, or technology or infrastructure type. For clarity here, we use the term service type because water source is often confused with the origin of the water (surface or ground) and technology often refers to the infrastructure or specific parts employed in the distribution of water. Data on water service type is collected at scale and monitored globally by WHO and UNICEF through their JMP. The JMP is tasked with monitoring progress towards MDG target 7.C and measures the proportion of people using an “improved” *vs*. “unimproved” drinking water source.

Two different dimensions are partially captured in service type classifications. The first concerns relative access, for example the JMPs top rung of “piped water at home” provides greater access than does a community source some distance from the household from which water is collected. This relative access dimension interacts with water quantity. The second concerns safety, since “improved sources”, when they are represented by community sources from which water is collected, are so defined based of their relative sanitary protection.

While service type is of direct relevance to policy and programming, there have been calls for more disaggregated reporting. For that purpose, it is noteworthy that service level classifications overlap extensively with the indicators safety and access, and, through the later, also with the indicator quantity.

### 3.2. Accessibility

The notion of access is complex. While it is most frequently captured through reference to single-journey collection time (itself assessed in different ways), it interacts with reliability (which determines “wasted journeys” when hardware is not functioning), water quality (for example, water quality decreases with handling and in storage), water quantity (for example, the longer the journey, the less water collection possible), and the characteristic of the collector (for example, children, the elderly and the disabled may collect smaller volumes in a single journey and be less able to make long collection journeys).

The large amount of time expended in walking to a water source and carrying water back to the household is recognized [44,45]. Women and children in low-income countries are the main water carriers and spend, on average, one hour per trip collecting water, with several trips required per day [44,45]. Time spent in walking to the water source is reported to be associated with lesser school attendance [46]. As travel time to the water source increases, there is also a reported decrease in water carriage to the household [22] and this can be associated with insufficient consumption and hydration [9]. Water carriage also has the potential to produce injury through musculoskeletal disorders and related disabilities [47].

Water quality deterioration is also associated with increased collection time since contamination may occur during collection, transport and storage [48,49,50,51]. This may explain the findings of recent research that points to less diarrheal disease as time to fetch water decreases [52].

Non-health effects of access levels are also substantive. An economic assessment found for every $1 investment in water was associated with benefits that could be valued at $2, and the main contributor to these economic benefits is the time saved with better access to services, which contributes 70% of the economic benefits [53,54].

### 3.3. Continuity and Reliability

“Continuity” refers to the ongoing stability (or lack) of a piped water supply or water source—typically assessed in terms of hours per day of service. It captures primarily anticipated interruptions in service—for example where a source dries up after a certain amount of collection and where piped supplies are available for known hours per day or days per week. “Reliability” is used to refer to the time which a point source or piped system is free from unplanned interruption due to breakdown or other causes. 

Lloyd and Bartram categorized the associated outcomes into four classes as year-round supply with no disruption, year-round service with daily variation; seasonal service variation; and compound (daily and seasonal) discontinuity [23]. Bartram categorized components of continuity as breakdown (unpredictable), regular daily discontinuity and regular seasonal discontinuity [26].

The lack of reliability and/or continuity in improved water sources can force households to search for other (potentially less safe) sources [55,56]. It also can contribute to contamination in piped systems [4,6]. Both are directly associated with contamination. A modeling exercise reported that the annual health benefits attributed to consumption of water from an improved supply would be lost because of raw water consumption arising from a few days breakdown [57]. This study suggested that reliability may be a reason for which studies of the impact of improved water quality on health have shown inconsistent results. In Europe, some 33% of waterborne disease outbreaks could be explained by problems in distribution [58] and pressure loss has been identified as a significant risk factor for sporadic diarrhea [57,59].

“Functionality” is related and a term now frequently used to mean the proportion of service points that are providing water at a given time, through cross-sectional survey.

### 3.4. Water Safety (Quality & Risk)

Inadequate water quality is associated with outbreak and endemic disease. The importance of water safety to health is widely recognized and well documented. Drinking water is an important transmission route for some agents of infectious diarrhea and other diseases [60], with the majority of the disease burden borne by children in developing countries [61]. Drinking water quality interventions are associated with a reduction in risk of diarrheal disease [62,63,64]. Improving water quality can decease parasitic infection and this can have an impact on school attendance and cognitive function in children [65,66]. Evidence from major cities across the United States in the late nineteenth and early twentieth century supports the conclusion that centralized chlorination and filtration of drinking water significantly reduced mortality rates [67].

Sanitary water safety, that is protection against fecal contamination, is assessed by measurement of indicators of fecal contamination in water samples (Section 3.4.1) and by an assessment of the adequacy of the sanitary protection of the source and system (Section 3.4.2). While chemical contamination of drinking water is also a health concern and some chemicals (notably fluoride and arsenic) may be associated with a substantive disease burden, microbial hazards dominate health concerns for water safety and are therefore our focus here.

As noted above, supply type and continuity/reliability are partial determinants of water safety [57,62]. While the MDG target refers to water safety, health benefits attributed to water safety accrue from the use of particular water service types over other less improved service types. For example, “improved” water sources have relatively greater protection from fecal contamination than unimproved sources [62,63]. Water safety is not measured by the JMP, rather use of an improved source is used as an indicator of it. The 2010 UN Resolution on The Human Right to Water and Sanitation recognizes that the right to safe and clean drinking water is a human right that is essential for the full enjoyment of life and all human rights [31].

#### 3.4.1. Measures of Drinking Water Quality

Water quality is assessed by measuring fecal indicator bacteria (FIB) in water. There are several FIB of which *E. coli* is most frequently measured. *E. coli* serves as an indicator of contamination rather than an index or risk or the degree of contamination. All indicators are imperfect, but other measures, such as intestinal enterococci (IE) may be preferable to *E. coli* as a predictor of risk in some circumstances. Water quality assessment is founded on sampling and because sampling is often undertaken at low intensity different sampling strategies may have a large impact on findings. In addition, water quality can change rapidly and shows marked seasonality. One-off or infrequent testing will, therefore, consistently and potentially substantively over-state the situation.

From first principles and studies in which human exposure has been assessed, it is evident that there is an exposure-response relationship between ingestion of fecally contaminated drinking water and outcomes such as infectious diarrhea. The exposure-response relationship is likely to be non-linear.

International norms for drinking-water safety are published by the WHO in the form of its *Guidelines for Drinking-water Quality*, which address risk assessment, based on microbiological water quality [24,68]. They provide a logarithmic scale of relative risk, based on counts of *E. coli* (Table 3). 

**Table 3:** WHO water quality risk levels (Reprinted from [24] with permission).

WHO water quality risk levels	
**Risk level**	*E. coli* **(CFU/100 mL)**
Conformity	<1
Low	1–10
Intermediate	11–100
High	101–1,000
Very High	>1,000

#### 3.4.2. Sanitary Risk

The WHO *Guidelines for Drinking-water Quality* promote assessment of sanitary risk for all drinking water supplies. Sanitary risk identifies sources of fecal contamination, potential pathways for contamination to reach water and measures to reduce contamination [68,69]. Specifically, surveillance of sanitary risk involves inspection of drinking water systems, the source of the drinking water, activities in the catchment area, transmission infrastructure, treatment plants, storage reservoirs and distribution systems [68]. A large cohort study in Canada found an association between sewage disposal and endemic infectious intestinal disease [70]. Research on Water Safety Plans, that incorporate sanitary risk factors, demonstrated positive health impacts on diarrheal disease reduction in Iceland [71].

#### 3.4.3. Combined Analysis

The combined analysis of sanitary inspection and water quality data can be used to identify the most important causes of and control measures for contamination [23,69]. Sanitary risk assessment can help to identify pathways for contamination and combining the two measures could be one way to assess safety. Research suggests that sanitary risk factors impact water quality. One study in Bangladesh, however, looked at tube well water quality contamination and found that it was not associated with a positive sanitary inspection score [72]. A study in Uganda of protected springs determined that some of the sanitary risk factors have a stronger association with contamination than others [73]. More research is needed in this area.

### 3.5. Quantity

The importance of sufficient water quantity for human health is documented [62,63]. Sufficient water for handwashing, hygiene, and bathing can reduce the spread of water-washed diseases including those spread through the fecal-oral route, as well as skin and eye diseases [28,74]. Sufficient water is critical for hydration and food preparation [9]. According to the World Health Organization (WHO), a minimum of 20 L per person per day is necessary and 100 L of water per person per day is optimal to ensure that consumption and hygiene needs are met [9]. Research suggests that sufficient water for productive household water uses can increase income generation for the household, and reduce poverty [21,36]. There is sizeable willingness to pay for water quantity improvements [75]. It is difficult, however, to disaggregate health impacts of water quantity interventions from water quality and accessibility interventions. Few randomized experiments have been done that look at the health impact of improving water quantity without accompanying changes in water quality or accessibility.

### 3.6. Equity, Non-Discrimination

Human rights research has explored the possibility of using equity as a metric for analyzing water services [76,77]. Data on household wealth indices as measured by Demographic and Health Surveys for example, and the level of service used could provide data to analyze the proportion of low-income households that receive each level of service. This can provide an indication of the equity of different types of service to the poor [78,79]. A human rights based approach has a foundation in non-discrimination; the Human Right to Water has empowered individuals to seek redress for rights violations; and international human rights law identifies individual rights-holders and their entitlements and corresponding duty bearers and their obligations [8]. Measuring improvement of services of certain groups, like the poorest or certain groups who are discriminated against, can help to measure improvements in equity.

A great deal of work has been done to measure inequality based on the principles of the Lorenze curve [80,81,82,83,84]; however, few metrics have been studied that measure equity in the delivery of domestic water services. Recent research has utilized the basic principles of human rights to develop an equity index, using rates of change compared to a benchmark rate rather than levels of achievement, to measure progressive realization for the human right to water and sanitation for each country [83]. Some research has been done to disaggregate data by wealth quintile [79]. Further research and international dialogue is needed on the advantages and disadvantages of different approaches to measuring equity, the potential for utilizing these methods at the household and community level, and the linkages between inequalities in water services and the impact on other indicators.

### 3.7. Cost and Affordability

Affordability as a criterion to measure access to services has been recognised for decades in various global and national water declarations and statements. In 2000, it was considered for adoption in the wording of the MDG target 7.C. In 2010, it was adopted by the United Nations General Assembly and the United Nations Human Rights Council as a normative concern for progressive realization of the human right to water [85]. The WHO *Guidelines for Drinking-water Quality* suggested, in 1997, that cost, defined as the amount spent by households on water services, is a factor that needs to be assessed when evaluating a water system [24].

The cost of a water service for households is influenced by water resource availability, construction costs of the water system, operation and maintenance costs, capital maintenance costs, expenditure on direct and indirect support [86]. Affordability as a global or national comparable criterion would need to capture how households contribute to all of these costs: in cash through tariffs, in cash through taxes and/or transfers and in kind, thought time spent on the construction and maintenance of the services, as well as any materials donated. Two aspects make affordability a complex criterion to assess: it does not explain fully willingness to pay for a service and there is no evidence base for what constitutes a “good enough” threshold. Willingness to pay for a particular service is influenced by a variety of factors beyond costs, including: quality of water, the continuity of the service provided, acceptability, and location [87,88]. 

Recent work has been done to review indicators proposed for measuring affordability of water, sanitation and hygiene (WSH) expenditure and evaluate them based on their validity; relevance and likely uptake; data requirements and availability; and resource needs for global monitoring [85]. The four main indicators evaluated were: (1) annual household WSH expenditure ÷ total annual household income; (2) WSH household capital expenditure ÷ total annual household income; (3) total WSH household expenditure ÷ total annual household income; (4) total financial and economic WSH household costs ÷ total annual household income [85]. While these indicators give a value for affordability of the service that can be compared across countries and regions with income levels available, they do not capture what water needs are met with the associated expenditure (e.g., water for drinking, hygiene, washing, garden, small business) or what value judgement can be made from such an analysis. Additional research and policy discussion is needed in this area to determine an appropriate indicator for affordability.

## 4. Water Service Delivery Indicators, Frameworks and Indices: Benefits and Challenges for Implementation, Policy, and Planning

Building on the above summary reviews of individual indicators, here we consider the issues surrounding implementation and use of the indicators in monitoring. Specifically, we analyze the challenges of a scale for each indicator, their inter-relationships and organization into monitoring frameworks, and the potential value of generating an index to summarily describe all indicators through a single metric.

### 4.1. A Scale for Each Indicator

In the cases of both service level and quality, there has been criticism of simple binomial categorization (pass/fail) and advocacy for more disaggregated and detailed information. Such information may be of value in policy and programming and in and of its own right. However, the focus of this paper is on assessment of relative value for public health, economic development and human rights fulfillment.

Logically, a scale for each indicator would culminate in some optimum—a level beyond which no further gains are anticipated. A scale would also need to fulfill two requirements: to represent meaningfully different degrees of adequacy (where adequacy refers to benefits to human health, economic development or human rights fulfillment) and to be reasonably measureable in ways that are comparable over time (to demonstrate changes) and over space (to enable comparison between communities states and countries).

For all of the indicators outlined above, however, there is very insufficient evidence for a scale that would reasonably meet these criteria. A possible exception may be water safety where a practical ceiling would be determined where repeated analyses do not detect *E. coli*, sanitary defects are absent, and scaling away from that in a manner similar to that described in Figure 1. While the capacity to implement such monitoring universally is not presently available, a sampling-based approach, similar to that used in nationally-representative household surveys but applied to sources and supply systems rather than households, may be achievable. In the case of access, there is some evidence for two transition points—one where water is available “on plot” and a second where the single journey collection time exceeds 30 min [22].

### 4.2. Monitoring Framework Approaches

In our above review summary of individual indicators, it is evident that there are substantive inter-dependencies among them. For example, the JMP service level indicator, in effect, combines a partial measure of sanitary protection (“improved” *versus* “unimproved” sources) with a partial measure of access (on-plot *versus* collected from shared source). Similarly “access” and continuity/reliability are both inter-dependent and both are likely determinants of quantity. Such relationship may be described though “framework approaches”. In effect four framework approaches can be found in the literature we describe:

- The first is the use of service levels as a simple summary description for which the JMP approach provides a well-recognized example [2]. The value of its simplicity in communication is evident as it maximizes comparability over time and space. It is also easily collected across geographical settings. Its weakness is the counter-face of that simplicity; it does not provide the more nuanced analysis of the factors that influence public health, economic development, and fulfillment of human rights.
- The second is a “level platform” of a set of indicators that typically include service type, safety, quantity, accessibility, reliability or continuity of service, affordability, and more recently equity and non-discrimination. The level platform enables all concerns to be monitored and additional concerns to be included at limited risk to integrity. Many individual indicators are inter-dependent, however, and subsequent indicators are likely to have lesser impact on overall outcome. However these same factors are its weaknesses.
- The third attempts to take some account of inter-dependencies and is based on the concept that a limited number of indicators can be meaningfully distinguished because certain indicators are derivatives of others [26]. The “value” of each indicator category can be discounted by lesser degrees of quality or continuity/reliability. In its derivation it assumes for example that access and continuity/reliability determine quantity and thus there is no merit in inclusion of quantity as a separate parameter. This is important at the national and international level where limited resources are available for monitoring. It is also useful to provide some logical relationship among indicators. The evident weakness is that while this derivation is logical, the evidence for the specific inter-dependencies is weak and may to a greater or lesser extent be context-specific in practice.
- The fourth takes a single measure from all indicators measured. For example, the IRC water service framework takes the worst performing indicator’s level to measure the service delivered [20]. The advantage is twofold: there is a single value for a service and this value can be disaggregated and the value for each indicator revealed. The disadvantages of taking the worst valued indicator are that detail on all other indicators measured is lost and their benefits are ignored.

### 4.3. Combination of Indicators into an Index

There is value in weaving together the evidence on indicators into an overall index whereby critical measurable indicators, directly associated with human health and well-being, track the quality of the service delivered. If a single value is desired from a group of indicators, IRC gives one approach to measure the service delivered; however, other forms of creating the index are defensible. They include: taking the 95th percentile “person” as a means of focusing on the lesser served [5], adding the levels of each indicator together using weights, similar to the Human Development Index, or a gap index whereby each indicator is assessed for how it deviates from a predetermined standard [89,90]. The methodological issues with an index include challenges with: aggregating different variables, determining specific weights for each variable (deciding on compensatory (scoring) or non-compensatory (ranking) weights), and validity of composites as policy tools [91,92]. In addition and important for monitoring, planning and policy, information is lost when a composite value is used to represent a group of indicators.

### 4.4. Evaluating Monitoring Frameworks

A purpose of adopting a monitoring framework is so that governments, international organizations, and communities can see change. If one has an index of indicators and scores for its components, a powerful analytical tool is created and operational research questions can be asked. If scales and scores can be agreed upon, we can begin to understand specific obstacles to water service delivery, and make comparisons cross country, state, or water service source so that action can be taken to address specific water service needs. A framework that includes information about specific indicators can aid in monitoring and evaluating water services, planning, and policy changes.

There are, however, challenges to adopting any one monitoring framework. They include: uniform adoption of measurable and comparable indicators, standardization of methods for data collection and comparability, and the identification of adequate resources to build capacity and facilitate data collection at scale. Agreement on measurable indicators is necessary so that they are consistently collected across households, schools, health centers, or at the level of the water system, and then aggregated at the community, municipal, provincial and country levels. In implementation of any one framework, further discussion is needed around what data collection is feasible in routine monitoring, what data can be collected in periodic surveys, and if household level monitoring is feasible for data collected on all indicators of if data collection could be at the community or water system level. Furthermore, relevant communities of practice need to be consulted and further tests of these frameworks conducted in the field are needed to ensure that they are scientifically valid, and contextually salient. Further research is also needed that evaluates the different monitoring frameworks. Criteria that are relevant to evaluating frameworks include: validity, reliability, relevance, data availability, and resource needs for data collection.

## 5. Conclusions

The underlying purposes of national and international monitoring of water for domestic purposes are in improving public health, securing economic development and fulfilling human rights. There is emerging consensus on factors (indicators) that contribute to achieving these purposes. There are, however, complex relationships among these factors and there is inadequate evidence with which to “score” the adequacy of different levels of each indicator, and questions as to whether scoring is achievable.

There are a few frameworks that may provide a basis for such monitoring, each with strengths and weaknesses. While the concept of an aggregate index has appeal, a sound scientific rationale is not available nor is their evidence that would enable its determination. Its communicative value is questionable and as is the case with any such aggregate, the benefits of simplicity must be weighed against loss of detail.

Further research and discussion is needed on the appropriate scale for each indicator, based on the health, economic and rights based benefits achieved. Post-2015 debates about what future water goals might include led to calls for a more rigorous and comprehensive monitoring system. Efforts to date have had little traction and the information with which to support scaling the different components is limited. In order to assess improvements, the subcomponents and how they individually and collectively impact health, economics and human rights need to be understood and measured as they explain the characteristics of a service. There are challenges to agreeing on certain indicators, timeframes for monitoring, and resource allocation to monitor and build capacity to enable data collection to occur.

We find that there is a general consensus on a short list of indicators and there is substantial research about the importance of each. However, more research is needed to understand how the indicators can be most effectively measured at the household or water system level and aggregated to the national and global levels. A few monitoring frameworks have been recommended; however, the application of the frameworks have not been fully explored in practice. Furthermore, while scales or ladders for each indicator have been recommended, they have not been sufficiently tested. In addition, while a substantive amount of evidence concerning the benefits of each factor exists, it is still incomplete. More work is needed to understand the health and economic benefits of varying levels of water quantity, the health and economic benefits of sanitary risk assessments, and the best indicators for equity and affordability. Furthermore, if a single composite metric is desired for an index of indicators, further research and dialogue is needed to determine the most appropriate approach.

As coverage with basic water supply is achieved, a more comprehensive monitoring of the quality of water service seems necessary. A water service framework that incorporates a small number of key indicators and an overall composite metric may provide a monitoring tool that is more comprehensive than currently exists, especially at the international level, and includes measurable indicators that speak to public health, economic development, and human rights concerns. By describing a given level of service in terms of a set of important indicators, and then grouping service levels into sequential and incrementally improved rungs on a water service delivery ladder, a useful tool for monitoring, planning and resource allocation can be created.
